# Supplementary material for: Marker-Assisted Pyramiding of CRa and CRd Genes to Improve the Clubroot Resistance of Brassica rapa
Source: Genes (Basel). 2022 Dec 19;13(12):2414. doi: 10.3390/genes13122414 (PMC9777773; doi:10.3390/genes13122414)
Supplement: Supplementary file 1 [file genes-13-02414-s001.zip › genes-2039795-supplementary.pdf]

Table S1. Molecular markers linked with *CRd* and *CRa* loci used for polymorphism screening

Marker	Primer-F	Primer-R
Yau96	ACGGCTAAACTATCAGCTGAGT	AGATCAAGAATTGGCCAGACA
Yau106	GGTCACCAATCGAAGCCTTT	GCATGCGGGTATACACATCT
Yau377	TTCACCGTTGTATTGGCGTG	GCTCTCCTCTAACCTGTTCCA
Yau389	TCAAGTGGAGAGGGTCAGTC	CAGTCACGAGGTCCCTAACA
Yau115	CCGTTTGGTTTCCCTTGCAA	GTTTAACACAGCAACAACAATGG
Yau135	GGACTTTAAATCGGCTGGACC	GAGGTCGACACAGAGAGATCA
Yau118	GAAGCTTCTGGATCACTGCG	ACAATAGCGAAATGGGCCAG
HC352b	CTTTATAATGGCTACTATTTA	TGCTCATGAGTGTATAACTA
CRa full length	ATGGATTTCTCTCTTTTCC	TTAACATGAGGGAGTTTCCAG
CRam	GCGTTTGAATCTTAGTCATTGCT	TCAAAGACTAACTCGGGAGCACTC

Table S2. The distribution of background markers on 10 linkage groups to CR5274

Linkage group	Number of markers	Linkage group length (Mb)	Physical distance (Mb)	Average interval (Mb)
A01	13	26.8	26.0	2.0
A02	9	26.9	25.9	2.9
A03	11	31.8	30.0	2.7
A04	7	19.3	18.9	2.7
A05	9	25.3	25.3	2.8
A06	10	25.2	25.2	2.5
A07	10	25.9	25.8	2.6
A08	9	20.8	20.8	2.3
A09	9	38.9	38.5	4.3
A10	8	16.4	14.3	1.8
Total	95	257.3	250.7	2.6
